# Not One Story: A Scoping Review Of Ethnic and Cultural Factors In Dementia Among Asian Americans

**DOI:** 10.1093/geroni/igaf122.3818

**Published:** 2025-12-31

**Authors:** Soohyun Park, Sylvia Wang, Arianna Patel, Eilis Reardon, Grace Jung, Anthony Mark, Ashley Paro

**Affiliations:** Tufts Medical Center, Boston, Massachusetts, United States; Widener University, Chester, Pennsylvania, United States; Tufts Medical Center, Boston, Massachusetts, United States; Tufts Medical Center, Boston, Massachusetts, United States; Tufts Medical Center, Boston, Massachusetts, United States; Tufts University School of Medicine, Boston, Massachusetts, United States; Tufts Medical Center, Boston, Massachusetts, United States

## Abstract

Asian Americans (AA) represent a rapidly growing and highly diverse population, yet dementia research often aggregates them into a single category. This practice limits culturally competent understanding and care for dementia in AA. This scoping review examines dementia terminology, risk and protective factors, cultural beliefs, caregiving practices, and cognitive screening tools across East AA (Chinese/Taiwanese, Japanese, Korean), South AA (Bangladeshi, Bhutanese, Indian, Maldivian, Nepalese, Pakistani, Sri Lankan), and Southeast AA (Burmese, Cambodian, Filipino, Hmong, Indonesian, Laotian, Malaysian, Thai, Vietnamese). Peer-reviewed articles published in English between 2000 and 2025 were identified through multiple databases, with findings synthesized narratively. Most studies focused on East AA, while South and Southeast AA remain markedly underrepresented. Across AA, terminology for dementia frequently carries negative connotations, though some East AA communities have begun adopting less stigmatizing language. Limited dementia literacy, misconceptions, and stigma are widespread, contributing to underdiagnosis and inadequate treatment. Caregiving is strongly shaped by familial and religious expectations, which often discourage institutional care and may result in social isolation, caregiver burden, and ethical dilemmas. Most validated cognitive screening tools are available for East AA. For South and Southeast Asian groups, only a few tools exist in their countries of origin, and some subgroups have no standardized assessments. This review provides a comprehensive summary of cultural nuances influencing dementia care and highlights critical gaps, particularly for South and Southeast AA. Future research should prioritize ethnically disaggregated data, culturally/linguistically tailored screening instruments, and partnerships with community and faith-based organizations to promote equitable dementia care for AA.

